# Contribution of Dental Amalgam to Urinary Mercury Excretion in Children

**DOI:** 10.1289/ehp.11013

**Published:** 2008-03

**Authors:** Ivo Iavicoli, Giovanni Carelli

**Affiliations:** Institute of Occupational Medicine, Catholic University of Sacred Heart, Roma, Italy, E-mail: gcarelli@rm.unicatt.it

Woods et al. (2007) studied a group of children (*n* = 507) who were exposed by inhalation to elemental mercury (Hg^0^) from dental amalgam fillings. In the study, 253 subjects were exposed, whereas the remaining 254 children, the control group, were exposed to composite resin. We consider the experimental design of their study to be adequate, but we do have questions about their methods of data handling and interpretation.

For example, we do not understand why instead of always using creatinine-adjusted Hg levels, they used—in some instances—unadjusted Hg levels. In fact, there is continuous alternation and exchange between the two biological concepts (i.e., between the unadjusted and the adjusted concentrations). There are at least three well-grounded and well-known reasons that creatinine adjustment is essential: *a*) urinary creatinine accounts for variations in 24-hr excretion (Aito et al. 1983); *b*) urinary creatinine adjustment reportedly reflects Hg blood levels (Smith et al. 1970) and possibly Hg body burden; and *c*) in the light of established knowledge, Hg blood levels reflect recent exposure (Piotrowski et al. 1975).

Accordingly, the lack of significance between the Hg levels (not adjusted for creatinine) of the amalgam and the control subgroups at year 7, the final year of the study by Woods et al. (2007), is probably a bias that is indicated by the disappearance of overlapping if creatinine adjustment had been performed, as suggested by Aitio et al. (1983) as long as 25 years ago. Also, because no adjusted data were reported for male and female levels, the impact of such an adjustment cannot be conjectured by the reader. Subsequently this prevents accurate evaluation of the Hg level trend over the years.

It should be pointed out that the data of Woods et al. (2007) do not allow us to extrapolate whether or not the exposed subgroup is in the steady state, because this condition depends on the time lag between urine collection and the last amalgam treatment(s). This limitation prevents an accurate interpretation of the decrease in urinary Hg levels over years.

Geller (1976) reported that Hg sulfide can coat Hg^0^, thereby slowing down the release of Hg vapor. Although no specific study has determined whether this is true for amalgams, we cannot exclude that Hg oxidation may yield Hg sulfide on the amalgam surface. Woods et al. (2007) speculated about the decrease in Hg urinary excretion over years, but they did not consider the possibility of sulfide formation. Moreover, they did not explain the decrease in Hg levels over time after year 2 but simply stated that “the rate of urinary [Hg] excretion exceeds the rate of [Hg] exposure from dental amalgam.” The formation of a thin film of Hg sulfide on amalgam surfaces could be an explanation, especially since the Hg body burden—and consequently Hg urinary levels—may be either in the steady state or at an increasing elimination rate because of the addition of new fillings.

Furthermore, we feel that the use of the term “dose–effect relationship” by Woods et al. (2007) is questionable. Also, it is not clear if the term “dose” refers to the number of additional amalgam fillings over the years or to the difference between means. Also, “effect” has a completely different meaning in toxicology. In this case, another term should be used to more accurately indicate the difference in two urinary Hg levels. In our opinion, “differential dose minus follow-up years” would be more appropriate in the text than “dose effect.”

Woods et al. (2007) stated that in children who received “up to 9 initial amalgam fillings, urinary Hg returned to pre-treatment value within one year,” but this statement is not clear because this trend applies only to children who received 0–4 amalgam fillings at baseline but not to the group that received 5–9 [Figure 4; Woods et al. (2007)].

Finally, Woods et al. (2007) omitted error bars from their Figure 4; SE or SD could have been easily calculated by the theory of error propagation and would probably have addressed the discussion more accurately, or at least would have tempered some conclusions, especially with regard to confirmation of the “whole-body biological half-time of Hg on the order of 60–70 days.” This half-time is correct but there is a large margin of uncertainty based on the experimental data.

In conclusion, although Woods et al. (2007) used a well-structured experimental design, their conclusions are not accurate because of their handling of the experimental results and their use of basic toxicology terminology.
